# 

**Published:** 1913-11-15

**Authors:** 


### PROMOTIONS AND VACANCIES.

Mr. R. B. N. Reade, fifth assistant medical officer at Hanwell Mental Hospital, has been appointed fourth assistant medical officer.

Vacancies at the London Homœopathic Hospital, Great Ormond Street and Queen Square, W.C., include those of full surgeon to the hospital, by the retirement of Mr. Knox Shaw, who has held the post for the past twenty-four years, and also a full physician, to replace Dr. MacNish, who has held appointments on the medical staff since 1895.

An Australian Appointment.—Dr. J. H. L. Cumpston, who has been acting as quarantine officer in Queensland, has been appointed to succeed Dr. W. P. Norris as Federal Quarantine Director, at a salary of £1,000 a year. Dr. Cumpston is M.B. and M.D. of the University of Melbourne. He also studied in Europe and took D.P.H. in London.

